# A photoactivatable Cre-loxP system for spatiotemporal genetic manipulation in mouse taste buds

**DOI:** 10.1016/j.jbc.2026.113085

**Published:** 2026-04-27

**Authors:** Yu Zuo, Kengo Horie, Yoshihiro Mitoh, Yasuhiro Yamada, Tomoka Takao, Takeshi Takarada, Shoichiro Kokabu, Ryusuke Yoshida

**Affiliations:** 1 Department of Oral Physiology, Graduate School of Medicine, Dentistry and Pharmaceutical Sciences, Okayama University, Okayama, Japan; 2 Faculty of Medicine, Dentistry and Pharmaceutical Sciences, Okayama University, Okayama, Japan; 3 Department of Molecular Pathology, Graduate School of Medicine and Faculty of Medicine, The University of Tokyo, Tokyo, Japan; 4 Department of Regenerative Science, Graduate School of Medicine, Dentistry and Pharmaceutical Sciences, Okayama University, Okayama, Japan; 5 Division of Biochemistry, Kyushu Dental University, Kitakyushu, Japan

**Keywords:** Cre-loxP, genetic manipulation, mouse, photoactivatable Cre, spatiotemporal, taste.

## Abstract

Conventional genetic approaches, including global gene KO and conditional KO strategies such as the Cre-loxP system, have some limitations arising from systemic effects or insufficient temporal resolution. The recently developed photoactivatable Cre (PA-Cre) system may have a potential to improve spatiotemporal control of gene manipulation. In this study, we established and validated the feasibility of the PA-Cre system using taste buds as a model. We generated TRE-PA-Cre:R26-rtTA/tdTomato mice to evaluate blue-light-induced Cre recombinase activity. Through systematic optimization of illumination parameters, we found that a single session of blue-light-illumination resulted in limited recombination efficiency, whereas a multisession illumination strategy markedly increased recombination efficiency. To further assess the utility of the PA-Cre system for gene KO, we generated TRE-PA-Cre:R26-rtTA:*Tas1r3*-flox mice and targeted a taste-related gene *Tas1r3*. Genomic DNA quantitative PCR and reverse transcription-quantitative PCR both showed partial reductions in *Tas1r3* at the DNA and mRNA levels, respectively. Behavioral assays further revealed a selective decrease in sensitivity to sweet and umami stimuli. Together, these findings demonstrate PA-Cre-mediated gene manipulation in taste buds and establish a practical optical activation paradigm, providing a high-spatiotemporal-resolution tool for investigating gene function in optically targeted regions.

Precise spatiotemporal genetic manipulation is particularly important to examine gene functions in the specific tissue. Conventional global KO approaches often result in embryonic lethality and lack spatiotemporal specificity (1, 2, 3, 4), leading to systemic effects that complicate phenotypic interpretation. To overcome these limitations, conditional gene targeting using the Cre-loxP system relies on bacteriophage P1 Cre recombinase to catalyze site-specific recombination between two 34-bp loxP sites flanking (“floxed”) a target DNA segment, with cell specificity determined by promoter- or enhancer-driven Cre expression (5). To further achieve temporal and cell-specific control of Cre activity, inducible Cre-loxP approaches such as tamoxifen-inducible CreERT2 have been developed. In the CreERT2 systems, Cre recombinase is fused to a mutant estrogen receptor ligand-binding domain and is sequestered in the cytoplasm by heat shock protein 90 kDa in the absence of tamoxifen. Upon binding of tamoxifen (or 4-hydroxytamoxifen), CreERT2 translocates to the nucleus and activates loxP-mediated recombination (6). Although this system provides temporal control of recombination (7), delayed activation kinetics and background activity limit their effective temporal resolution.

To improve spatiotemporal regulation of gene manipulation, a photoactivatable Cre (PA-Cre) system has been developed. PA-Cre employs blue-light-induced dimerization of split Cre fragments and has been shown to support rapid, reversible, and low-background recombination with high temporal precision (8, 9). One such approach is the magnet-based split-Cre system in which light-responsive protein domains (nMag and pMag) heterodimerize upon blue-light-illumination, thereby restoring Cre recombinase activity (9). Recent studies have developed PA-Cre systems and demonstrated the usefulness of PA-Cre for spatiotemporal control of gene manipulation using cell based assays (10, 11). These systems have been applied to *in vivo* studies. For example, in TRE-PA-Cre:ROSA26-tdTomato mice, hydrodynamic tail vein injection of tTA expression vectors followed by external noninvasive blue-light-illumination induced Cre-loxP recombination in the liver (12). PA-Cre knock-in mice showed light-induced recombination monitored in multiple organs of reporter mice, including the brain, heart, liver, spleen, thymus, skin, muscle, kidney, and lung, and enabled site-specific recombination by spot irradiation or long-term irradiation using an implanted wireless LED (13). PA-Cre was also used to induce Cre-loxP recombination in the mouse brain and liver through AAV-based delivery, hydrodynamic tail vein injection, and conditional mouse lines (14). However, a functional KO model mediated by PA-Cre activation has not been tested yet.

Taste buds consist of 50 to 100 tightly packed taste cells located within the fungiform (FP), circumvallate (CV), and foliate papillae (FoP). These papillae exist in the different location, FP in the anterior and CV and FoP in the posterior tongue (Fig. 1*A*). Importantly, anterior and posterior taste fields differ not only in location but also in structure, innervation, and sensory roles in taste processing (15). Thus, spatially restricted gene manipulation by PA-Cre may be clearly evaluated by comparing different taste fields, such as FP and CV taste buds. Taste cells are morphologically and functionally classified into four types (16, 17, 18). Type I glial-like supporting cells play critical roles in peripheral taste detection and signal transmission (19, 20, 21). Type II cells are responsible for sweet, umami, and bitter taste detection whereas type III cells mediate sour taste (22, 23, 24). Type IV basal progenitor cells differentiate to other taste-cell types (18, 25). In addition, taste cells undergo continuous and rapid turnover, with approximately ∼10 to 11% replaced each day and an average lifespan of 10 to 14 days (26, 27), although cell type specific longevities have been reported in mouse taste buds (28). Such cellular heterogeneity and the rapid turnover of taste cells necessitate genetic tools that enable precise spatial and temporal control of gene expression. Regarding taste sensitivity, sweet and umami responses differ between the chorda tympani and glossopharyngeal nerve, which innervate anterior and posterior taste fields, respectively (29). However, *Tas1r3*, which encodes a component of the sweet/umami taste receptor, is expressed in multiple anatomically distinct taste papillae, including FP, CV, and FoP (30), suggesting differential roles of *Tas1r3* gene among FP, CV, and FoP. Partial, region-restricted gene KO may reveal contributions of specific gene in each taste field to taste functions that would be masked when all taste fields are simultaneously altered in a global KO model.

**Figure 1:**
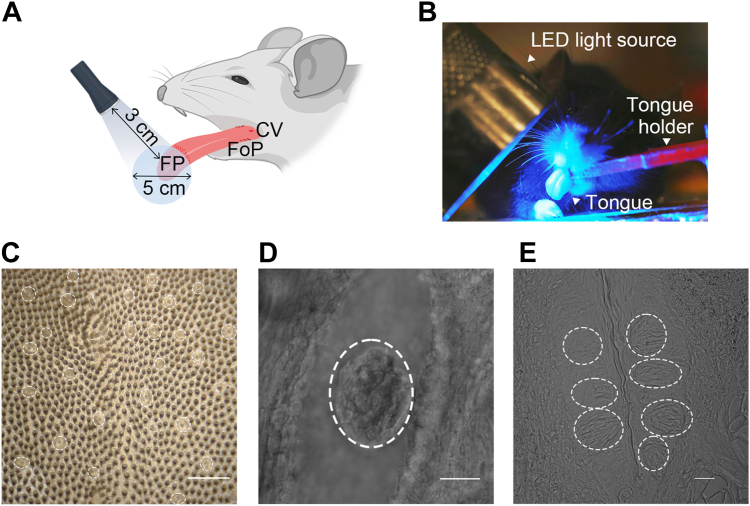
**Localized *blue-light-illumination* of the anterior tongue and transmitted light views of FP and CV samples.***A*, schematic illustration showing that *blue-light-illumination* was limited to the anterior tongue region containing FP, whereas the posterior tongue region containing CV and FoP lay outside the illuminated field. *B*, actual photograph of *blue-light-illumination* during the experiment. *C*, low-magnification transmitted light image of the anterior tongue surface showing the FP region. The scale bar represents 125 μm. *D*, higher-magnification transmitted light image of an FP taste bud. The scale bar represents 20 μm. *E*, transmitted light image of the CV region. *Dotted line* indicates the outline of a taste bud. The scale bar represents 20 μm. FP, fungiform; CV, circumvallate; FoP, foliate papillae.

In addition to the spatial heterogeneity within taste buds, taste receptors and other taste-related genes are also expressed in extraoral organs. For example, *Tas1r3* is not restricted to taste buds but is also expressed in multiple other tissues, including the hippocampus and intestinal epithelium (31). In the brain, *Tas1r3* expression has been associated with functions such as cognition and social behavior (32). In the intestine, *Tas1r3* has been implicated in glucose sensing and the regulation of hormone secretion such as GLP-1 release in response to luminal glucose (33, 34). Consequently, global *Tas1r3* deletion may produce behavioral phenotypes that reflect combined effects across multiple tissues, complicating the interpretation of taste-bud-intrinsic functions. In addition, field-specific viral gene manipulation across anatomically distinct taste papillae remains technically challenging. Current *in vivo* AAV-mediated gene transfer has been demonstrated mainly in FP taste cells after submucosal tongue injection, suggesting that controlled comparison among different taste fields is still difficult (35).

The present study addresses these challenges by evaluating the applicability of the PA-Cre system for anatomically restricted and time-defined gene manipulation in mouse taste buds, optimizing key experimental parameters, and demonstrating its practical utility through targeted gene deletion where conventional global KO approaches are confounded by extraoral effects and region-restricted viral manipulation remains technically challenging.

## Results

### PA-Cre enables blue-light-dependent tdTomato induction in taste buds *in vivo*

**Figure 2:**
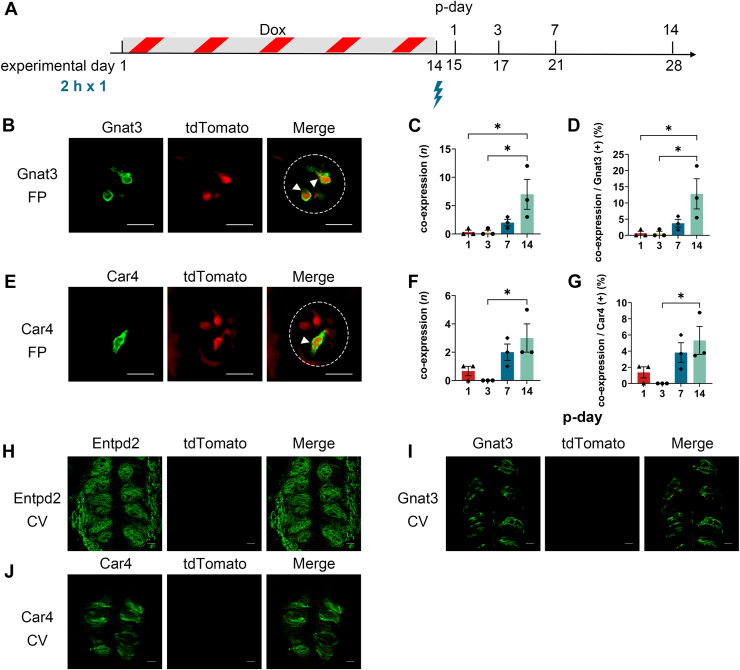
**Effects of p-day on PA-Cre recombination efficiency in FP taste buds of TRE-PA-Cre:R26-rtTA/tdTomato mice.***A*, experimental timeline for PA-Cre recombination. Dox was provided in drinking water at 2 mg/ml for 14 consecutive days (experimental days 1–14) to induce rtTA-dependent PA-Cre expression. Mice received a single *blue-light-illumination* (2 h) on experimental day 14. The expression of tdTomato in taste buds was analyzed at p-day 1, 3, 7, or 14 (experimental day 15, 17, 21, or 28). *B*, representative immunofluorescence images of FP taste buds for Gnat3 at p-day 14. *C* and *D*, the number (*C*) and proportion (*D*) of coexpressing FP taste buds (Gnat3 and tdTomato) among Gnat3-positive taste buds. *E*, representative immunofluorescence images of FP taste buds for Car4 at p-day 14. *F* and *G*, the number (*F*) and proportion (*G*) of coexpressing FP taste buds (Car4 and tdTomato) among Car4-positive taste buds. *H*–*J*, TRE-PA-Cre;R26-rtTA/tdTomato mice administrated Dox and subjected blue-light-illumination (2 h, one session) on the anterior tongue. Nonilluminated CV taste buds were analyzed at p-day 14 to assess whether PA-Cre activation remained spatially restricted. Markers: (*H*) Entpd2, (*I*) Gnat3, and (*J*) Car4. *Dotted line* indicates the outline of a taste bud. *Arrowheads* indicate taste cells coexpressing the marker and tdTomato. The scale bar represents 20 μm. The results of one-way ANOVA and *post hoc* Tukey’s HSD test are summarized in Table S1. ∗*p* < 0.05. p-day, postillumination period; PA-Cre, photoactivatable Cre; FP, fungiform; TRE, tetracycline response element; Dox, doxycycline; rtTA, reverse tetracycline-controlled transactivator; HSD, honestly significant difference.

A blue-light-illumination setup was used to deliver localized illumination to the anterior tongue of anesthetized mice (Fig. 1, *A* and *B*). Illumination was limited to the FP-containing anterior tongue, whereas CV and FoP were outside the illuminated field. To see the reporter expression, we used peeled tongue epithelium containing FP taste buds (Fig. 1, *C* and *D*) and slice preparation of CV taste buds (Fig. 1*E*). To determine whether blue-light-illumination of the anterior tongue induces PA-Cre-dependent reporter expression *in vivo* in FP taste buds, we analyzed tdTomato expression at 1, 3, 7, and 14 days after a single illumination session with 2 h illumination duration under doxycycline (Dox) induction in tetracycline response element (TRE)-PA-Cre:R26-reverse tetracycline-controlled transactivator (rtTA)/tdTomato mice (Fig. 2*A*). To assess the general applicability of the optimized parameters, we evaluated PA-Cre recombinase efficiency in taste cell subpopulations with distinct lifespans, including type II and type III taste cells. We used Gnat3 as a type II cell marker and Car4 as a type III cell marker. Confocal imaging showed that subsets of Gnat3-positive (type II) and Car4-positive (type III) taste cells in the FP expressed tdTomato following blue-light-illumination (Fig. 2, *B* and *E*). Both the number and the proportion of FP taste buds coexpressing Gnat3 or Car4 with tdTomato were significantly increased in a time-dependent manner during the postillumination period (p-day) (Fig. 2, *C*, *D*, *F*, and *G*; one-way ANOVA, *p* < 0.05, Table S1). These results indicate that tdTomato expression in FP taste buds of TRE-PA-Cre:R26-rtTA/tdTomato mice gradually increased following a single blue-light-illumination.

**Figure 3:**
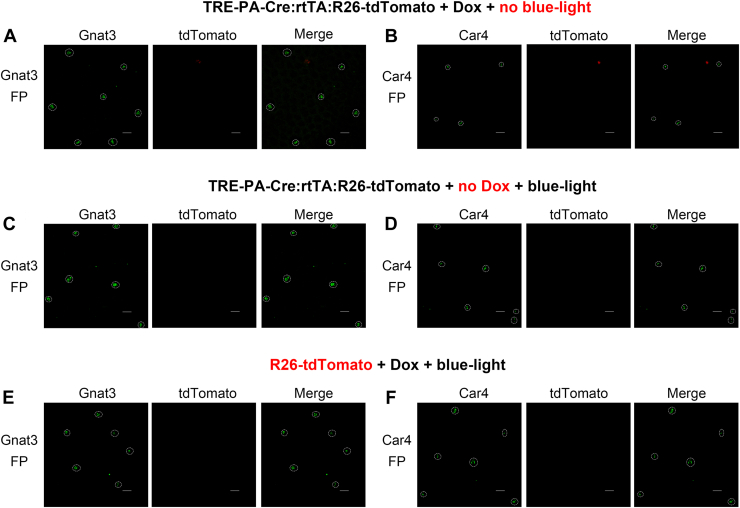
**Negative-control validation of background PA-Cre activity in mouse taste buds.***A* and *B*, TRE-PA-Cre;R26-rtTA/tdTomato mice administrated Dox, but not subjected to *blue-light-illumination*. FP taste buds were analyzed at p-day 14. Markers: (*A*) Gnat3 and (*B*) Car4. *C* and *D*, TRE-PA-Cre;R26-rtTA/tdTomato mice not administrated Dox but subjected to *blue-light-illumination* (2 h, one session) on the anterior tongue. Illuminated FP taste buds were analyzed at p-day 14. Markers: (*C*) Gnat3 and (*D*) Car4. *E* and *F*, R26-tdTomato mice administrated Dox and subjected to *blue-light-illumination* (2 h, one session). Illuminated FP taste buds were analyzed at p-day 14. Markers: (*E*) Gnat3 and (*F*) Car4. Across all control conditions and markers, no tdTomato-positive taste buds were detected in taste buds. *Dotted line* indicates the outline of a taste bud. The scale bar represents 20 μm. PA-Cre, photoactivatable Cre; TRE, tetracycline response element; Dox, doxycycline; rtTA, reverse tetracycline-controlled transactivator; FP, fungiform; p-day, postillumination period.

We next examined whether reporter induction occurred in the absence of individual induction components. Taste buds are present not only in the anterior part of the tongue (FP) but also on the posterior part of the tongue (such as CV). Because blue-light-illumination was restricted to the anterior tongue, we hypothesized that taste buds in the CV would not express tdTomato due to the absence of blue-light-illumination. We analyzed CV taste buds of TRE-PA-Cre:R26-rtTA/tdTomato mice under the induction condition (2 h illumination, single session, p-day 14), as our p-day analysis showed that tdTomato expression increased markedly by p-day 7 to 14. Confocal imaging revealed no detectable tdTomato fluorescence in CV taste buds under this condition (Fig. 2, *H*–*J*). These results indicate that the PA-Cre fusion protein does not exhibit spontaneous dimerization or detectable leak activity in the absence of blue-light-illumination. Furthermore, we confirmed spatially restricted activation of PA-Cre by blue-light-illumination. We also confirmed FP taste buds of TRE-PA-Cre:R26-rtTA/tdTomato mice treated with Dox but without blue-light-illumination. Under these conditions, FP taste buds showed no tdTomato expression (Fig. 3, *A* and *B*). To test Dox dependency, we omitted Dox treatment while applying blue-light-illumination (2 h illumination, single session) and analyzed FP taste buds at p-day 14 (Fig. 3, *C* and *D*). No tdTomato expression was detected in FP taste buds, suggesting that Dox is required for expression of PA-Cre in this mouse line. Finally, we confirmed that R26-tdTomato mice alone did not exhibit any leak expression of tdTomato in FP taste buds (Fig. 3, *E* and *F*). Together, the absence of background reporter expression demonstrates the high stringency of this system and supports the utility of PA-Cre for precise spatiotemporal control of gene manipulation in the mouse tongue *in vivo*.

### Parameter optimization for PA-Cre activation in taste buds

**Figure 4:**
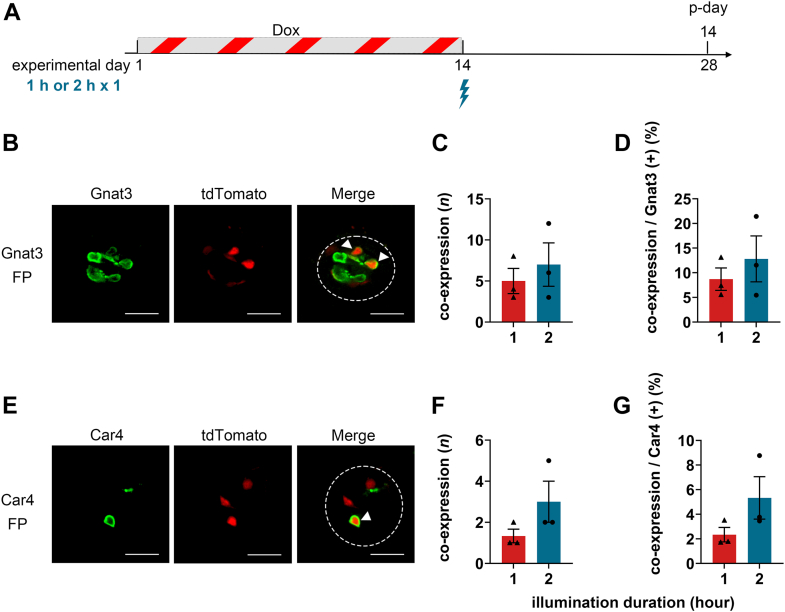
**Effects of illumination duration on PA-Cre recombination efficiency in FP taste buds of TRE-PA-Cre:R26-rtTA/tdTomato mice.***A*, experimental timeline for PA-Cre recombination. Dox was administrated for 14 consecutive days (experimental days 1–14). Mice received a single *blue-light-illumination* session on experimental day 14, with illumination duration set to either 1 h or 2 h. The expression of tdTomato in taste buds was analyzed at p-day 14 (experimental day 28). *B*, representative immunofluorescence images of FP taste buds for Gnat3. *C* and *D*, the number (*C*) and proportion (*D*) of coexpressing FP taste buds (Gnat3 and tdTomato) among Gnat3-positive taste buds. *E*, representative immunofluorescence images of FP taste buds for Car4. *F* and *G*, the number (*F*) and proportion (*G*) of coexpressing FP taste buds (Car4 and tdTomato) among Car4-positive taste buds. *Dotted line* indicates the outline of a taste bud. *Arrowheads* indicate taste cells coexpressing the marker and tdTomato. The scale bar represent 20 μm. The results of unpaired Student's *t* test are summarized in Table S2. *p* > 0.1. PA-Cre, photoactivatable Cre; FP, fungiform; TRE, tetracycline response element; rtTA, reverse tetracycline-controlled transactivator; Dox, doxycycline; FP, fungiform; p-day, postillumination period.

We next tested whether illumination duration affects PA-Cre recombinase efficiency. The illumination duration was reduced from 2 h to 1 h and reporter expression in FP taste buds was assessed at p-day 14 (Fig. 4*A*). Statistical analyses of both the number and the proportion of FP taste buds coexpressing Gnat3 or Car4 with tdTomato demonstrated no significant differences between the 1 h and 2 h illumination durations, although the values tended to be higher with the 2 h illumination (Fig. 4, *C*, *D*, *F*, and *G*; Student’s *t* test, *p* > 0.1, Table S2). These results indicate that illumination duration alone is not a major optimization parameter for enhancing PA-Cre recombinase efficiency. Therefore, a 2 h illumination duration was used as the standard condition in subsequent experiments.

**Figure 5:**
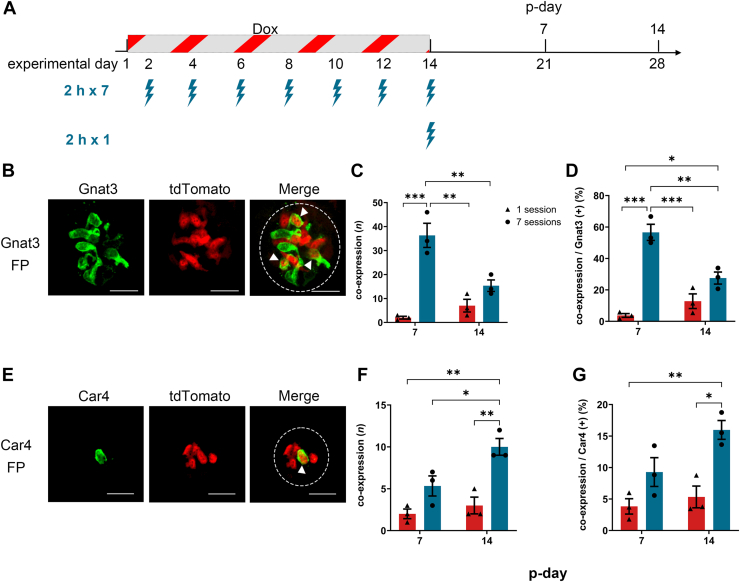
**Combined effects of p-day and illumination frequency on PA-Cre recombination efficiency in FP taste buds of TRE-PA-Cre:R26-rtTA/tdTomato mice.***A*, experimental timeline for PA-Cre recombination. Dox was administrated for 14 consecutive days (experimental days 1–14). Mice received seven illumination sessions with a fixed illumination duration of 2 h. Seven illumination sessions were delivered at 2-day intervals (experimental days 2, 4, 6, 8, 10, 12, and 14). The expression of tdTomato in taste buds was analyzed on p-days 7 and 14 (experimental days 21 and 28). *B*, representative immunofluorescence images of FP taste buds for Gnat3 at p-day 7. *C* and *D*, the number (*C*) and proportion (*D*) of coexpressing FP taste buds (Gnat3 and tdTomato) among Gnat3-positive taste buds. *E*, representative immunofluorescence images of FP taste buds for Car4 at p-day 7. *F* and *G*, the number (*F*) and proportion (*G*) of coexpressing FP taste buds (Car4 and tdTomato) among Car4-positive taste buds. *Dotted line* indicates the outline of a taste bud. *Arrowheads* indicate taste cells coexpressing marker and tdTomato. The scale bar represents 20 μm. The results of two-way ANOVA are summarized in Table 1 and *post hoc* Tukey’s HSD test are summarized in Table S3. ∗*p* < 0.05; ∗∗*p* < 0.01; ∗∗∗*p* < 0.001. p-day, postillumination period; PA-Cre, photoactivatable Cre; FP, fungiform; TRE, tetracycline response element; rtTA, reverse tetracycline-controlled transactivator; Dox, doxycycline; HSD, honestly significant difference.

Following a single illumination session, 12.81 ± 4.65% of Gnat3-positive taste buds and 5.33 ± 1.72% of Car4-positive taste buds expressed reporter at p-day 14 (Fig. 2 and 4). In order to enhance reporter expression in FP taste buds, we next tested a repetitive illumination strategy by increasing the number of illumination sessions while keeping the illumination duration constant at 2 h. The experimental paradigm is illustrated in Fig. 5*A*. For Gnat3-positive type II cells, both the number and the proportion of FP taste buds coexpressing tdTomato were significantly increased in seven illumination sessions compared to a single session at p-day 7 (Fig. 5, *B*–*D*, Tables 1 and S3). However, comparison between p-days 7 and 14 under the seven-session condition revealed that both the number and the proportion of coexpressing FP taste buds were significantly decreased at p-day 14 compared to p-day 7 (Fig. 5, *C* and *D*, Tables 1, and S3). For Car4-positive type III cells, both the number of and the proportion of coexpressing FP taste buds were significantly increased with seven illumination sessions compared to a single session at p-day 14 (Fig. 5, *E*–*G*, Tables 1, and S3). However, in contrast to Gnat3, the number of coexpressing FP taste buds was significantly higher at p-day 14 than at p-day 7 (Fig. 5*F*, Tables 1, and S3). These results suggest that increasing the number of illumination sessions effectively enhances reporter expression in FP taste buds. However, the temporal dynamics of reporter expression might differ between type II and III taste cells under the multisession illumination condition.

**Table 1:** Two-way ANOVA results for parameter optimization for PA-Cre Activation (Fig. 5)

Stained (primary antibody)	Variable	p-day		Frequency		Interaction	
		F	p	F	P	F	p
Gnat3	coexpressing taste buds (Fig. 5*C*)	6.640	0.033	47.216	<0.001	17.533	0.003
	coexpression ratio (Fig. 5*D*)	6.260	0.037	71.490	<0.001	22.766	0.001
Car4	coexpressing taste buds (Fig. 5*F*)	8.500	0.019	28.265	0.001	3.559	0.096
	coexpression ratio (Fig. 5*G*)	5.615	0.045	21.669	0.002	2.252	0.172

Frequency, illumination frequency.

### Validation of PA-Cre-mediated *Tas1r3* deletion

**Figure 6:**
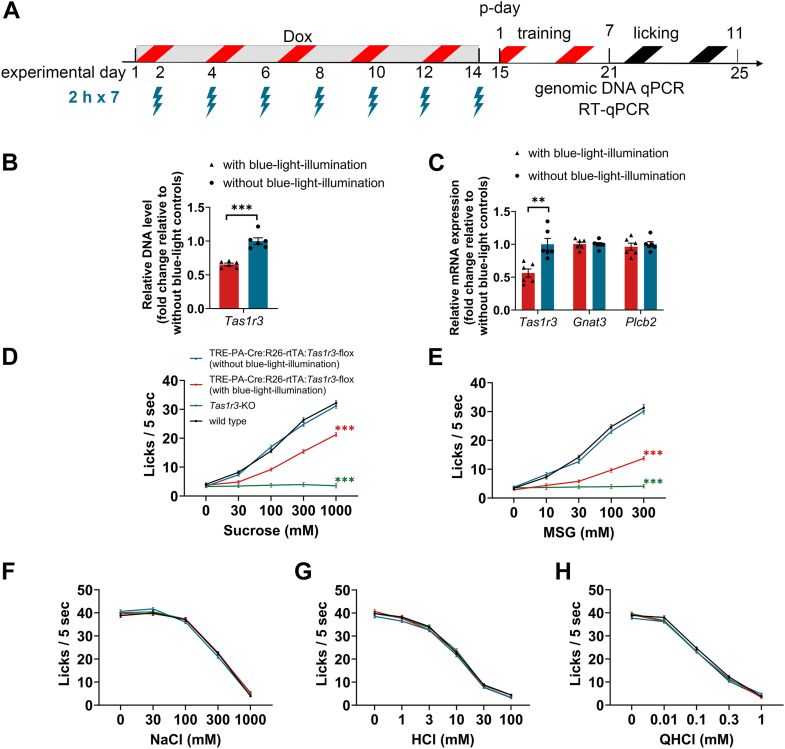
**Molecular and behavioral validation of PA-Cre-mediated *Tas1r3* deletion.***A*, experimental timeline for PA-Cre recombination. Dox was administrated for 14 consecutive days (experimental days 1–14). Mice received seven illumination sessions with a fixed illumination duration of 2 h. Illumination sessions were delivered at 2-day intervals (experimental days 2, 4, 6, 8, 10, 12, and 14). Training for the short-term lick test begins from p-day 1 (experimental day 15) and lick test for each tastant starts from p-day 7 (experimental day 21). Genomic DNA qPCR and RT-qPCR were performed at p-day 7 (experimental day 21). *B*, comparison of relative genomic DNA signal in FP taste buds between TRE-PA-Cre:R26-rtTA:*Tas1r3*-flox mice with (*red*) and without (*blue*) *blue-light-illumination* (each *N* = 6). Genomic DNA signal in the floxed *Tas1r3* region was evaluated relative to a reference amplicon in exon 6 and is presented relative to the nonilluminated control group. *G*, relative mRNA expression of *Tas1r3*, *Gnat3*, and *Plcb2* in FP taste buds between TRE-PA-Cre:R26-rtTA:*Tas1r3*-flox mice with (*red*) and without (*blue*) application of *blue-light-illumination* (each *N* = 6). Expression levels were normalized to *Gapdh* and are presented as fold change relative to the nonilluminated control group. *D*–*H*, short-term lick responses to sweet (sucrose, *D*), umami (MSG, *E*), salty (NaCl, *F*), sour (HCl, *G*), and bitter (QHCl, *H*) tastants in WT (*black*), *Tas1r3*-KO (*green*), TRE-PA-Cre:R26-rtTA:*Tas1r3*-flox mice with (*red*) and without (*blue*) application of *blue-light-illumination* (each *N* = 9). Unpaired Student’s *t*-tests for genomic DNA qPCR and RT-qPCR are summarized in Tables S4 and S5, respectively. The results of repeated two-way ANOVA for the short-term lick test are summarized in Table 2 and *post hoc* Tukey’s HSD test are summarized in Table S6. ∗∗∗*p* < 0.001 in (*B*). ∗∗*p* < 0.01 in (*C*). ∗∗∗*p* < 0.001 in (*D* and *E*) (*versus* other three types of mice, *post hoc* Tukey HSD test). PA-Cre, photoactivatable Cre; Dox, doxycycline; p-day, postillumination period; qPCR, quantitative PCR; RT-qPCR, reverse transcription-qPCR; FP, fungiform; MSG, monosodium glutamate; QHCl, quinine-HCl.

Based on the optimized parameter analysis, p-day 7, 2 h illumination duration, and seven illumination sessions appeared to maximize reporter expression in type II taste cells of TRE-PA-Cre:R26-rtTA/tdTomato mice (Figs. 2, 4, and 5). Under this regimen, 56.60 ± 5.12% of Gnat3-positive taste buds expressed reporter (Fig. 5*D*). We next assessed the consequences of PA-Cre-mediated *Tas1r3* deletion using the experimental design shown in Fig. 6*A*. For molecular analyses, FP taste buds were collected from TRE-PA-Cre:R26-rtTA:*Tas1r3*-flox mice with or without blue-light-illumination for genomic DNA quantitative PCR (qPCR) and reverse transcription quantitative PCR (RT-qPCR).

As an initial genomic validation, we performed genomic DNA qPCR analysis on FP taste buds from TRE-PA-Cre:R26-rtTA:*Tas1r3*-flox mice with or without blue-light-illumination (Fig. 6*B*). Relative genomic DNA signal, normalized to a reference amplicon located in exon 6 outside the deleted region and presented as fold change relative to the nonilluminated control group, was significantly reduced in illuminated mice compared with nonilluminated controls (Student’s *t* test, *p* < 0.001, Table S4). These results support partial recombination/deletion of the floxed *Tas1r3* allele at the DNA level.

We next examined whether this genomic change was accompanied by altered *Tas1r3* mRNA expression (Fig. 6*C*). Relative *Tas1r3* mRNA expression, normalized to *Gapdh*, was significantly reduced in illuminated mice compared with nonilluminated controls (Student’s *t* test, *p* < 0.01, Table S5). In contrast, relative expression levels of downstream signaling components of *Tas1r3*, such as *Gnat3* and *Plcb2*, were not significantly different between illuminated and nonilluminated mice. These results indicate that blue-light-induced PA-Cre activation selectively reduces relative *Tas1r3* mRNA expression in FP taste buds of TRE-PA-Cre:R26-rtTA:*Tas1r3*-flox mice without detectable effects on other key taste signaling genes.

We then asked whether these molecular changes were associated with altered taste behavior. Behavioral responses were compared among TRE-PA-Cre:R26-rtTA:*Tas1r3*-flox mice with or without blue-light-illumination, C57BL/6J WT and conventional *Tas1r3*-KO mice. TRE-PA-Cre:R26-rtTA:*Tas1r3*-flox mice with or without blue-light-illumination were subjected to the behavioral assay described in Figure 6*A*, and other controls were directly assessed without Dox and blue-light-illumination. In the short-term lick test, TRE-PA-Cre:R26-rtTA:*Tas1r3*-flox mice without blue-light-illumination exhibited responses to all tastants (sucrose, monosodium glutamate (MSG), HCl, NaCl, and quinine-HCl(QHCl)) comparable to those of WT mice (Fig. 6, *D*–*H*, and Table 2). In contrast, *Tas1r3*-KO mice showed abolished responses to sucrose and MSG, but responded similarly to WT mice for other tastants (Tables 2, and S6). TRE-PA-Cre:R26-rtTA:*Tas1r3*-flox mice subjected to blue-light-illumination exhibited a significant reduction in lick responses to sucrose and MSG compared with both WT and TRE-PA-Cre:R26-rtTA:*Tas1r3*-flox mice without blue-light-illumination (Tables 2, and S6). However, responses to sucrose and MSG in TRE-PA-Cre:R26-rtTA:*Tas1r3*-flox mice with blue-light-illumination remained significantly greater than those observed in *Tas1r3*-KO mice (Tables 2, and S6). These results demonstrate that PA-Cre-mediated *Tas1r3* deletion selectively impairs sweet and umami taste responses without broadly affecting other taste modalities.

**Table 2:** Repeated two-way ANOVA results for the short-term lick test (Fig. 6)

Taste	Mice		Concentration		Interaction	
	F	p	F	p	F	p
sucrose (Fig. 6*D*)	262.575	<0.001	824.719	<0.001	104.716	<0.001
MSG (Fig. 6*E*)	223.390	<0.001	546.027	<0.001	89.123	<0.001
NaCl (Fig. 6*F*)	0.411	0.746	2709.148	<0.001	1.851	0.047
HCl (Fig. 6*G*)	1.900	0.149	3034.883	<0.001	0.762	0.691
QHCl (Fig. 6*H*)	1.144	0.346	3702.874	<0.001	1.996	0.045

Together, these results support effective but partial PA-Cre-mediated deletion of *Tas1r3* under the present experimental conditions.

## Discussion

This study established and validated spatiotemporal gene manipulation in adult mouse taste buds by using PA-Cre system. To date, functional studies have largely relied on global or conditional KO models. However, global gene KO could lead to systemic effects outside the taste buds that confound the attribution of taste-bud-specific phenotypes (32). In contrast, CreERT2 approaches are limited by tamoxifen pharmacokinetics, including delayed induction (commonly on the order of 12–48 h) (36), which restricts temporal precision. Moreover, a recent study demonstrated that tamoxifen induced a transcriptional switch from proliferation to differentiation, thereby introducing a potential confounding effect in taste research (37). The PA-Cre system enables blue-light-dependent activation of Cre recombinase with low background activity and superior temporal control (minimum time window: ∼1 h) compared with the CreERT2 approach (9). Through systematic optimization of illumination parameters, we demonstrate that PA-Cre is an effective tool for manipulating gene expression in adult mouse taste buds with precise spatiotemporal control.

We found that reporter expression in FP taste buds of TRE-PA-Cre:R26-rtTA/tdTomato mice was time dependent following blue-light-illumination (Fig. 2). Reporter expression was minimal at p-day 1 to 3 and increased markedly by p-day 7 to 14. These results indicate a lag between blue-light-illumination and detectable tdTomato signal at the taste-bud level. This delay likely reflects both the time required for the accumulation of tdTomato protein (38), and the time required for Cre-mediated recombination. The latter may be influenced by the speed and efficiency of Cre dimerization, nuclear translocation, and recombination. Previous work has shown that recombination activity using the PA-Cre system peaks at 24 to 48 h after blue-light exposure (9). However, our results showed that tdTomato expression in FP taste buds had not yet reached a level sufficient for cell detection at p-day 1 to 3. This difference might be derived from the promoter strength, with tdTomato expression in FP taste buds becoming detectable approximately seven days after blue-light-illumination. In contrast, illumination duration (1 h and 2 h) did not significantly affect PA-Cre activity in FP taste buds of TRE-PA-Cre:R26-rtTA/tdTomato mice. PA-Cre could be triggered by brief (∼30 s) blue-light-illumination, and Magnet dimers persist with a photocycle half time of approximately 1.8 h. Thus, the gain from extending illumination from 1 h to 2 h might be limited (9). Consistent with this, Kishi *et al.* (2021) reported low recombination efficiency after 1 h of illumination, but observed further increases when illumination was extended to 6 h or longer (39). Based on these findings, we speculate that illumination durations exceeding 2 h may be required to achieve a more substantial increase in recombination efficiency. However, in our experimental setup, prolonged illumination may affect animal physiology due to extended anesthesia and tongue desiccation.

PA-Cre activation is strongly dependent on blue-light, eliciting reporter gene expression only upon blue-light-illumination, with minimal to no background expression in the dark, thereby ensuring precise light-dependent activation (9, 12, 13, 14). In our study, PA-Cre dependent reporter expression was not induced in the absence of blue-light-illumination (Fig. 3). Moreover, even when blue-light was applied to the anterior tongue, PA-Cre dependent reporter expression was not observed in the posterior tongue (Fig. 2, *H*–*J*). This spatially restricted activation of PA-Cre may be useful for examining regional differences between the anterior and posterior tongue. Furthermore, this approach could be applied to achieve organ-specific gene manipulation in future studies by applying blue-light to a specific organ. Oral sweet ingestion can trigger a variety of physiological responses. For example, stimulation of *Tas1r3* expressed in both taste buds and pancreatic β cells has been suggested to contribute to pattern of insulin secretion (40, 41). In global KO models, however, responses mediated by both sites are simultaneously disrupted, making it difficult to determine the relative contribution of each tissue. In contrast, the PA-Cre system offers an approach to distinguish the relative contributions of taste buds and pancreatic β cells to each phase of insulin secretion, thereby helping to distinguish the physiological roles of extraoral taste receptors from those of taste-bud-intrinsic signaling. In our system, PA-Cre expression is Dox-dependent, as rtTA must bind Dox to activate PA-Cre transcription, thereby PA-Cre is expressed only in the presence of Dox and resulting in minimal expression in its absence (42, 43). If a specific gene promoter would be used to induce rtTA expression, organ-gene specific gene manipulation could be achieved. Thus, combining the PA-Cre and rtTA systems enables more precise spatiotemporal control of gene manipulation.

Regarding illumination sessions, a single illumination session resulted in approximately 5∼13% of Gnat3-and Car4-positive taste buds expressed reporter (Fig. 2 and 4), indicating relatively low PA-Cre efficiency in FP taste buds. Previous study demonstrated that multisessions enhance recombination efficiency in COS-7 cells (9), suggesting that repetitive illumination improves PA-Cre mediated recombination. In our study, increasing the number of illumination sessions to seven significantly increased reporter expression (Fig. 5). These data indicate the effectiveness of repeated blue-light-illumination. Given the continuous taste cell turnover (average lifespan ∼10–14 days) and their time-dependent maturation (18, 44), repetitive illumination likely increased detectable reporter expression in FP taste buds by extending the effective time windows of photoactivation across ongoing taste cell renewal and maturation. With a single illumination session, only a limited number of taste cells is exposed at a given time, and subsequent cell loss, together with the delay in tdTomato expression, may reduce the number of tdTomato positive cells. In contrast, delivering multiple illumination sessions at 2-day intervals repeatedly recruits newly available taste cells into the activation window, thereby increasing the cumulative number of recombined taste cells.

Notably, the optimal p-day was cell type dependent. Under the condition of seven illumination sessions, reporter expression in Gnat3-positive taste buds was greater at p-day 7 than at p-day 14. On the other hand, reporter expression in Car4-positive taste buds was greater at p-day 14 than p-day 7 (Fig. 5). These differences likely reflect distinct cell longevity between Gnat3-positive (type II) and Car4-positive (type III) taste cells. Previous studies have demonstrated that lineage turnover differs among cell types, with type II cells having a half-life of ∼8 days and type III cells ∼22 days (28). For type II taste cells, repeated blue-light-illumination likely increases the number of tdTomato positive cells initially. However, due to their short lifespan, these labeled cells gradually disappeared. Consequently, at p-day 14, the loss of tdTomato-positive cells exceeds the generation of newly labeled cells, resulting in a reduced number of double positive cells at this time point. In contrast, tdTomato positive type III taste cell has a longer lifespan and therefore retained over the course of the experiment, leading to an increase in the number of double positive cells from 7 to 14 days after blue-light-illumination.

As a final step of technical validation, we demonstrated that PA-Cre-mediated manipulation of *Tas1r3* under the optimized conditions produced consistent molecular and behavioral changes (Fig. 6). Genomic DNA qPCR and RT-qPCR both showed significant, but partial reductions in *Tas1r3*, supporting partial recombination/deletion of the floxed *Tas1r3* allele after blue-light-illumination. This partial and region-restricted recombination likely explains why the behavioral phenotype of blue-light-illuminated TRE-PA-Cre:R26-rtTA:*Tas1r3*-flox mice was weaker than that of *Tas1r3*-KO mice. In addition to the incomplete reduction of *Tas1r3* expression in FP taste buds, residual *Tas1r3* expression in nonilluminated taste fields, including the CV, FoP, palate, and pharyngeal taste buds, may contribute to the remaining lick responses to sucrose and MSG. Consequently, blue-light-illumination TRE-PA-Cre:R26-rtTA:*Tas1r3*-flox mice retained a substantial population of functional sweet- and umami-responsive taste cells across these fields, which were likely sufficient to preserve partial sweet and umami sensitivity.

Compared with conventional global KO approaches, the major advantage of PA-Cre-mediated recombination is not maximal deletion efficiency *per se*, but the ability to achieve anatomically restricted and time-defined gene manipulation while avoiding potential confounding effects arising from *Tas1r3* loss in extraoral tissues such as the intestine or brain (32), thereby enabling more stringent spatiotemporal gene manipulation. At the same time, the relatively low recombination efficiency of this system (∼max 57% in this study) remains a limitation and should be improved in future studies, for example by developing a more potent PA-Cre system or using a stronger light source to achieve more complete recombination in illuminated taste cells.

In conclusion, the PA-Cre system provides an effective tool for spatiotemporal gene manipulation in adult mouse taste buds, combining precise blue-light-dependent activation. Although repeated illumination sessions substantially enhanced recombination efficiency, the remaining limitations highlight the need for further development and optimization to achieve near-complete recombination in taste cells in future studies.

## Experimental procedures

### Animal and ethics

All animal procedures were approved by and performed in accordance with the Institutional Animal Care and Use Committees of Okayama University and the Institute of Medical Science, The University of Tokyo, Japan. Adult mice (>8 weeks, both sexes) were used. Test mice were singly housed under a 12 h red LED light (LT-FLE900R-HL, 12w, 623 nm, Ohm Electric.)/12h dark cycle (lights on 08:00–20:00; ambient temperature 25 °C ± 1 °C; relative humidity ∼50–60%) with standard chow (MF, Oriental Yeast) and water ad libitum. Red LED room light was used for housing and behavioral testing to mitigate the background recombination of PA-Cre. WT mice were purchased from CLEA Japan. Original stock of *Tas1r3*-KO (29) and R26-tdTomato mice (45) was obtained from Dr Robert F. Margolskee (Monell Chemical Senses Center) and Jackson laboratory, respectively.

To generate *Tas1r3*-flox mice (*Tas1r3* flox/flox), a loxP sequence was inserted 49 bp upstream of exon 1 and 75 bp downstream of exon 5 in the *Tas1r3* gene, respectively. A guide RNA (gRNA) targeting this region was designed, and the gRNA, Cas9 mRNA, and donor DNA containing the loxP sequence were microinjected into fertilized C57BL/6J embryos. The resulting offspring were genotyped by PCR, and heterozygous mice carrying the loxP sequence upstream of exon 1 (*Tas1r3* loxP/+) were obtained. Subsequently, fertilized eggs derived from these loxP knock-in mice were used to introduce a second loxP sequence downstream of exon 5 (in the intron 5 region). A gRNA targeting this site was designed, and microinjection was performed in the same manner. Genomic DNA extracted from tail biopsies of the obtained pups was analyzed by PCR and Sanger sequencing to confirm the precise insertion of the loxP sites upstream of exon 1 and downstream of exon 5. All procedures for mouse generation were performed by SetuRotech Co, Ltd.

TRE-PA-Cre:R26-rtTA/tdTomato mice were generated by crossing TRE-PA-Cre mice (12), R26-rtTA mice (46, 47), and R26-tdTomato mice. For generation of TRE-PA-Cre:R26-rtTA:*Tas1r3*-flox mice, TRE-PA-Cre mice, R26-rtTA mice, and *Tas1r3*-flox mice were crossed.

### Dox application

To induce rtTA-dependent expression of PA-Cre, Dox (Combi-Blocks; Code SS-7516; CAS 10592-13-9) was provided in drinking water at 2 mg/ml for 14 consecutive days (experimental days 1–14). Dox water was prepared fresh every 2 days, protected from light, and supplied ad libitum in a water bottle. One % Sucrose was added to improve palatability. Animals were maintained under red LED room light only during this period to prevent unintended photoactivation.

### Illumination protocol

Mice were first anesthetized by intraperitoneal injection with a 100 μl/10 g body weight anesthetic cocktail (Dorbene, 1.9 mg/1.9 ml; Dormicum, 10 mg/2 ml; Vetorphale, 5 mg/2.5 ml; to 25 ml with sterile water) (Dorbene vet; Kyoritsu Seiyaku; NVAL ID 11573; CAS 86347-15-1) (Dormicum; Sandoz; YJ code 1124401A1060; CAS 59467-70-8) (Vetorphale; Meiji Animal Health; Code B5-VETLI; CAS 58786-99-5). After anesthesia, the mouse was placed and secured on a 37 °C heating pad attached to a similarly sized box fixed on the experimental table. A 470 nm LED light source (LED470-3W; OptoCode) was held by a light-holding arm, which was clamped to the side edge of the experimental table. The mouse tongue was then gently pulled out with forceps. Photoactivation was subsequently performed with the light source positioned ∼3 cm above the exposed anterior region of the tongue, producing an illumination field of ∼5 cm in diameter (Fig. 1, *A* and *B*). The entire setup, including the mouse and illumination apparatus, was covered with a light-shielding box during illumination to maintain a dark environment and prevent unintended photoactivation. Each illumination session consisted of 30 min continuous illumination followed by a 5 min pause, during which the tongue was moistened with sterile saline. At the end of illumination, mice received Antisedan (Nippon Zenyaku Kogyo; NVAL ID 2622; CAS 104075-48-1) by intraperitoneal injection dosed by body weight (200 μl per 30*g*). Illumination duration was defined as the total illumination time per session (1 h = two cycles; 2 h = four cycles). Illumination frequency was defined as either a single session (illumination at experimental day 14 of Dox treatment) or seven sessions delivered at 2-day intervals (illumination at experimental days 2, 4, 6, 8, 10, 12, and 14 of Dox treatment). P-day was defined as the interval from the end of illumination to tissue collection/analysis; under the seven-session procedure, p-day was referenced to the final illumination session. Optical power was specified by the manufacturer, and no overt tissue damage or behavioral abnormalities were observed after blue-light-illumination.

Illumination parameters were optimized by varying one factor at a time while holding the others constant. First, to optimize the p-day under the 1-session condition, mice received a single illumination session with the fixed illumination duration (2 h), and the expression of tdTomato in taste buds was analyzed at p-day 1, 3, 7, or 14 (correspond to experimental day 15, 17, 21, or 28) (Fig. 2*A*). Second, to test the illumination duration, mice received a single illumination session (1 or 2 h) and the expression of tdTomato in taste buds was analyzed at p-day 14 (experimental day 28) (Fig. 4*A*). Third, to optimize illumination frequency, mice received seven illumination sessions with the fixed illumination duration (2 h). The expression of tdTomato in taste buds was assessed at p-day 7 or 14 (experimental day 21 or 28) (Fig. 5*A*).

### Immunofluorescence staining and confocal imaging

Immunofluorescence staining was performed with minor modifications from a previously described method (48). Adult TRE-PA-Cre:R26-rtTA/tdTomato mice or R26-tdTomato mice were euthanized in a CO_2_ chamber. For FP, the anterior tongue was excised and rinsed with Tyrode’s solution. Then, 100 μl Tyrode’s solution containing elastase (Elastine Products; Code EC134; CAS 39445-21-1) (0.25 mg/ml) was injected between the epithelial layer and the underlying connective/muscle tissue to facilitate epithelial separation. After 15 min, the epithelium was gently peeled, pinned on a Sylgard-coated dish, and fixed in 4% paraformaldehyde (PFA, Nacalai Tesque; Code 26126–25; CAS 30525-89-4) at 4 °C for 25 min. For CV, the posterior tongue was dissected, fixed in 4% PFA at 4 °C for 1 h, cryoprotected in 15% sucrose for 1 h, followed by 2 h incubation in 30% sucrose at 4 °C, embedded in OCT compound (Sakura Finetek; Code 4583), and cryosectioned at 10 μm. FP epithelium and CV sections were washed in Tris-buffered saline containing 0.1% Tween-20, blocked with Blocking One-P (Nacalai Tesque; Code 05999-84) for 1 h at RT, and incubated overnight at 4 °C with primary antibodies: Entpd2 (sheep IgG; R&D Systems; RRID: AB_10572702; working 1:400), Gnat3 (goat IgG; Aviva Systems Biology; RRID: AB_10882823; working 1:200), and Car4 (goat IgG; R&D Systems; RRID: AB_2070332; working 1:400). After Tris-buffered saline containing 0.1% Tween-20 washes, samples were incubated with Alexa Fluor 488-conjugated secondary antibodies (donkey antigoat IgG (H + L), Thermo Fisher Scientific; RRID: AB_2534102; working 1:200; or donkey antisheep IgG (H + L), Abcam; RRID: AB_2801320; working 1:200), washed, and mounted with Fluoromount-G medium (Thermo Fisher Scientific; REF 00–4958–02). Confocal images were acquired using confocal laser scanning microscope (Carl Zeiss,; model LSM 780) (10 × /40 × ; 488/568 nm) with sequential channel acquisition, and channels were merged in ZEN software (Carl Zeiss). Only taste cells with distinct borders were manually counted; counting was performed without blinding.

### Experimental design, sample size, and quantification

FP and CV were harvested from mice and processed as described above at p-day 1, 3, 7 or 14. To assess background/leak, four control conditions were analyzed (*N* = 3 mice per group per marker; total 24 mice: 12 for Gnat3 immunostaining and 12 for Car4 immunostaining). For testing spatial specificity, blue-light was applied only to the anterior tongue of TRE-PA-Cre:R26-rtTA/tdTomato mice treated with Dox. tdTomato expression was then examined in both the illuminated anterior FP and the nonilluminated posterior CV to determine whether PA-Cre activation remained spatially restricted (Fig. 2). To test illumination dependency, FP taste buds of TRE-PA-Cre:rtTA:R26-tdTomato treated with Dox but without blue-light-illumination were analyzed (Fig. 3, *A* and *B*). For the test of dox dependency, FP taste buds of TRE-PA-Cre:rtTA:R26-tdTomato treated with blue-light-illumination but without Dox were analyzed (Fig. 3, *C* and *D*). To validate nonspecific tdTomato expression, FP taste buds of R26-tdTomato mice treated with Dox and blue-light-illumination were analyzed (Fig. 3, *E* and *F*).

For parameter testing, p-day was varied (p-day 1, 3, 7 or 14; 1 session; 2 h; *N* = 3 mice per condition per marker; total 24 mice), illumination duration was compared (1 *versus* 2 h; 1 session; p-day 14; *N* = 3 mice per condition per marker; total 12 mice), and illumination frequency was compared (1 *versus* 7 sessions; 2 h; p-day 7 or 14; *N* = 3 mice per condition per marker; total 24 mice). Overlapping datasets were shared across experiments for statistical comparisons. All analyses treated each mouse as an independent biological replicate. For each FP, we quantified Gnat3-or Car4-positive, tdTomato-positive, and coexpressing taste buds, the corresponding ratios. A taste bud was classified as positive if it contained at least one taste cell with distinct borders, indicating Gnat3-or Car4-positivity.

### Genomic DNA qPCR

Genomic DNA qPCR in taste bud cells was performed using FP taste buds collected from TRE-PA-Cre:R26-rtTA:*Tas1r3*-flox mice with (*N* = 6) or without blue-light-illumination (*N* = 6) at p-day 7 (Fig. 6*A*). Genomic DNA was extracted using the NucleoSpin Tissue kit (Takara Bio Inc.; Code U0952A/product code 740952.50). qPCR was performed on a QuantStudio 1 (Thermo Fisher Scientific) using THUNDERBIRD Next SYBR qPCR Mix (TOYOBO). Reactions were run in duplicate. Each mouse was treated as one biological replicate. Amplification specificity was confirmed by melt-curve analysis showing a single peak without detectable primer-dimer formation. Relative quantification of genomic DNA signal was performed using the 2^-ΔΔCt^ method. The amplicon within the floxed *Tas1r3* region (using *Tas1r3*-KO primers) was normalized to a reference amplicon located in exon 6 outside the deleted region (using *Tas1r3*-WT primers), and values are presented as fold change relative to the nonilluminated control group. Primer sequences are listed in Table 3.

**Table 3:** Primers for qPCR

Primer	Forward	Reverse	Product
*Tas1r3*-KO	ACAAGTGGTGGTGCTGTTTG	TGTCAGCCAAGACTCACTGG	112 bp
*Tas1r3*-WT	CAAGGCCCTGCAGTGCA	AGGCCTTAGGTGGGCATAATAGGA	92 bp
*Tas1r3*	TCAAGGCACAAGGGGACTAC	GAACAAACCAAGGGGTGAGA	128 bp
*Gnat3*	AGGGCATCTGAATACCAGCTCAA	CTGATCTCTGGCCACCTACATCAA	196 bp
*Plcb2*	GGCTACCTCCTAAAGCACGA	ACAGGAACTGCCCAGAGATG	123 bp
*Gapdh*	TGTGTCCGTCGTGGATCTGA	TTGCTGTTGAAGTCGCAGGAG	150 bp

### RT-qPCR

RT-qPCR in taste bud cells was performed as previously described (48). FP taste buds were collected from TRE-PA-Cre:R26-rtTA:*Tas1r3*-flox mice with (*N* = 6) or without blue-light-illumination (*N* = 6) at p-day 7 (Fig. 6*A*). Total RNA was purified with the FastGene RNA Premium Kit (NIPPON Genetics; Code FG-81050/FG-81250). RNA samples were quantified using NanoDrop Lite spectrophotometer (Thermo Fisher Scientific). First-strand cDNA was synthesized with ReverTra Ace qPCR RT Kit (TOYOBO; Code FSQ-101). Unless otherwise stated, qPCR conditions, reaction settings, and amplification specificity assessment were the same as those described above for genomic DNA qPCR. Relative quantification of gene expression was conducted using the 2^−ΔΔCt^ method. mRNA expression level was normalized to *Gapdh*. Primer sequences are listed in Table 3.

### Short-term lick test

Behavioral lick responses to various tastants were recorded as described previously (49). Eight weeks old WT (*N* = 9), *Tas1r3*-KO (*N* = 9), and TRE-PA-Cre:R26-rtTA:*Tas1r3*-flox mice with and without blue-light-illumination (each *N* = 9), housed in individual cages, were used as experimental subjects. On day 1 of training (p-day 1), each animal was water-deprived for 12 h and then placed in the test cage and given free access to distilled water (DW) during the 1 h session. Days 2 to 6 (p-days 2–6) comprised training sessions, during which animals were trained to drink DW on an interval schedule consisting of 5-s periods of DW presentation alternating with 10-s intertrial intervals. From day 7 (p-day 7), the numbers of licks for each taste solution and DW were counted during the first 5 s after the animal’s first lick, using a lick meter (Yutaka Electronics) (Fig. 6*A*). Tastants used in short-term lick test are: sucrose (Nacalai Tesque; Code 30404–45; CAS 57-50-1) (30–1000 mM) + 1 mM QHCl (Nacalai Tesque; Code 29910-32; CAS 6119-47-7), MSG (Nacalai Tesque; Code 16914-05; CAS 142-47-2) (10–300 mM) + 1 mM QHCl, NaCl (Nacalai Tesque; Code 31320-05; CAS 7647-14-5) (30–1000 mM), HCl (Nacalai Tesque; Code 37314-15; CAS 7647-01-0) (1–100 mM) and QHCl (0.01–1 mM). On each test day, mice were given test solutions with concentrations of descending order (from highest concentration to DW for sucrose and MSG) or ascending concentration order (from DW to highest concentration for NaCl, HCl, and QHCl) in the first trial then randomized order in subsequent trials. Each concentration was tested more than three times per animal and mean lick counts were used for statistical analysis.

### Statistical analysis

Statistical analyses were performed in R (v4.5.2; R Foundation for Statistical Computing), and graphs were generated using GraphPad Prism (GraphPad Software). Normality was assessed using the Shapiro-Wilk test together with Q-Q plots. Data are presented as mean ± SEM. For immunofluorescence staining measurements of illumination duration, genomic DNA qPCR and RT-qPCR data, two-group comparisons were performed using two-tailed unpaired Student’s *t* test. For immunofluorescence staining measurements of the p-day, differences among three or more groups were assessed by one-way ANOVA. For immunofluorescence staining measurements of the p-day and illumination frequency, two-way ANOVA was used to evaluate main effects and interactions. Short-term lick test data were analyzed by two-way repeated-measures ANOVA to evaluate main effects and interactions. Post hoc Tukey’s honestly significant difference test was used for multiple-comparisons (Welch correction was applied when variances were unequal). The significance threshold was *p* < 0.05. For immunofluorescence staining, each data point represents the measurement from 1 mouse (one biological replicate) (Fig. 2, 4, and 5). For genomic DNA qPCR and RT-qPCR, each data point represents 1 mouse (biological replicate) (*N* = 6) (Fig. 6). For the short-term lick test, each data point represents the group value at each concentration (*N* = 9 mice per group) (Fig. 6). No data points were excluded unless stated.

## Data availability

All data reported in this paper will be shared with the lead contact upon request.

## Supporting information

This article contains supporting information (statistical results).

## Conflict of interest

The authors declare that they have no conflicts of interest with the contents of this article.

## References

[1] Kreidberg J.A., Sariola H., Loring J.M., Maeda M., Pelletier J., Housman D. (1993). WT-1 is required for early kidney development. Cell.

[2] Zeitlin S., Liu J.P., Chapman D.L., Papaioannou V.E., Efstratiadis A. (1995). Increased apoptosis and early embryonic lethality in mice nullizygous for the Huntington's disease gene homologue. Nat. Genet..

[3] Frank K.M., Sekiguchi J.M., Seidl K.J., Swat W., Rathbun G.A., Cheng H.L. (1998). Late embryonic lethality and impaired V(D)J recombination in mice lacking DNA ligase IV. Nature.

[4] Götz J., Probst A., Ehler E., Hemmings B., Kues W. (1998). Delayed embryonic lethality in mice lacking protein phosphatase 2A catalytic subunit calpha. Proc Natl Acad Sci U. S. A..

[5] Lewandoski M. (2001). Conditional control of gene expression in the mouse. Nat. Rev. Genet..

[6] Kim H., Kim M., Im S.K., Fang S. (2018). Mouse Cre-LoxP system: general principles to determine tissue-specific roles of target genes. Lab Anim. Res..

[7] Feil R., Brocard J., Mascrez B., LeMeur M., Metzger D., Chambon P. (1996). Ligand-activated site-specific recombination in mice. Proc. Natl. Acad. Sci. U. S. A..

[8] Kennedy M.J., Hughes R.M., Peteya L.A., Schwartz J.W., Ehlers M.D., Tucker C.L. (2010). Rapid blue-light-mediated induction of protein interactions in living cells. Nat. Methods.

[9] Kawano F., Okazaki R., Yazawa M., Sato M. (2016). A photoactivatable Cre-loxP recombination system for optogenetic genome engineering. Nat. Chem. Biol..

[10] Taslimi A., Zoltowski B., Miranda J.G., Pathak G.P., Hughes R.M., Tucker C.L. (2016). Optimized second-generation CRY2-CIB dimerizers and photoactivatable Cre recombinase. Nat. Chem. Biol..

[11] Duplus-Bottin H., Spichty M., Triqueneaux G., Place C., Mangeot P.E., Ohlmann T. (2021). A single-chain and fast-responding light-inducible Cre recombinase as a novel optogenetic switch. Elife.

[12] Takao T., Hiraoka Y., Kawabe K., Yamada D., Ming L., Tanaka K. (2020). Establishment of a tTA-dependent photoactivatable Cre recombinase knock-in mouse model for optogenetic genome engineering. Biochem. Biophys. Res. Commun..

[13] Yoshimi K., Yamauchi Y., Tanaka T., Shimada T., Sato M., Mashimo T. (2021). Photoactivatable Cre knock-in mice for spatiotemporal control of genetic engineering *in vivo*. Lab. Invest..

[14] Morikawa K., Furuhashi K., de Sena-Tomas C., Garcia-Garcia A.L., Bekdash R., Klein A.D. (2020). Photoactivatable Cre recombinase 3.0 for in vivo mouse applications. Nat. Commun..

[15] Kumari A., Mistretta C.M. (2023). Anterior and posterior tongue regions and taste papillae: distinct roles and regulatory mechanisms with an emphasis on hedgehog signaling and antagonism. Int. J. Mol. Sci..

[16] Iwata S., Yoshida R., Ninomiya Y. (2014). Taste transductions in taste receptor cells: basic tastes and moreover. Curr. Pharm. Des..

[17] Taruno A., Nomura K., Kusakizako T., Ma Z., Nureki O., Foskett J.K. (2021). Taste transduction and channel synapses in taste buds. Pflugers Arch..

[18] Finger T.E., Barlow L.A. (2021). Cellular diversity and regeneration in taste buds. Curr. Opin. Physiol..

[19] Wilson C.E., Lasher R.S., Salcedo E., Yang R., Dzowo Y., Kinnamon J.C. (2025). Death in the taste bud: engulfment of dying taste receptor cells by glial-like type I cells. Glia.

[20] Park G.Y., Lee G., Yoon J., Han J., Choi P., Kim M. (2025). Glia-like taste cells mediate an intercellular mode of peripheral sweet adaptation. Cell.

[21] Rodriguez Y.A., Roebber J.K., Dvoryanchikov G., Makhoul V., Roper S.D., Chaudhari N. (2021). "Tripartite Synapses" in taste buds: a role for type I glial-like taste cells. J. Neurosci..

[22] DeFazio R.A., Dvoryanchikov G., Maruyama Y., Kim J.W., Pereira E., Roper S.D. (2006). Separate populations of receptor cells and presynaptic cells in mouse taste buds. J. Neurosci..

[23] Yoshida R., Miyauchi A., Yasuo T., Jyotaki M., Murata Y., Yasumatsu K. (2009). Discrimination of taste qualities among mouse fungiform taste bud cells. J. Physiol..

[24] Horie K., Wang K., Huang H., Yasumatsu K., Ninomiya Y., Mitoh Y. (2025). Dual functions of SNAP25 in mouse taste buds. J. Physiol..

[25] Ren W., Lewandowski B.C., Watson J., Aihara E., Iwatsuki K., Bachmanov A.A. (2014). Single Lgr5- or Lgr6-expressing taste stem/progenitor cells generate taste bud cells ex vivo. Proc. Natl. Acad. Sci. U. S. A..

[26] Beidler L.M., Smallman R.L. (1965). Renewal of cells within taste buds. J. Cell Biol..

[27] Farbman A.I. (1980). Renewal of taste bud cells in rat circumvallate papillae. Cell Tissue Kinet..

[28] Perea-Martinez I., Nagai T., Chaudhari N. (2013). Functional cell types in taste buds have distinct longevities. PLoS One.

[29] Damak S., Rong M., Yasumatsu K., Kokrashvili Z., Varadarajan V., Zou S. (2003). Detection of sweet and umami taste in the absence of taste receptor T1r3. Science.

[30] Nelson G., Hoon M.A., Chandrashekar J., Zhang Y., Ryba N.J., Zuker C.S. (2001). Mammalian sweet taste receptors. Cell.

[31] Yoshida R., Ninomiya Y. (2024). Mechanisms and functions of sweet reception in oral and extraoral organs. Int. J. Mol. Sci..

[32] Martin B., Wang R., Cong W.N., Daimon C.M., Wu W.W., Ni B. (2017). Altered learning, memory, and social behavior in type 1 taste receptor subunit 3 knock-out mice are associated with neuronal dysfunction. J. Biol. Chem..

[33] Margolskee R.F., Dyer J., Kokrashvili Z., Salmon K.S., Ilegems E., Daly K. (2007). T1R3 and gustducin in gut sense sugars to regulate expression of Na+-glucose cotransporter 1. Proc. Natl. Acad. Sci. U. S. A..

[34] Jang H.J., Kokrashvili Z., Theodorakis M.J., Carlson O.D., Kim B.J., Zhou J. (2007). Gut-expressed gustducin and taste receptors regulate secretion of glucagon-like peptide-1. Proc. Natl. Acad. Sci. U. S. A..

[35] Taruno A., Kashio M., Sun H., Kobayashi K., Sano H., Nambu A. (2017). Adeno-associated virus-mediated gene transfer into taste cells in vivo. Chem Senses.

[36] Hayashi S., McMahon A.P. (2002). Efficient recombination in diverse tissues by a tamoxifen-inducible form of Cre: a tool for temporally regulated gene activation/inactivation in the mouse. Dev. Biol..

[37] Oura N., Koyanagi-Matsumura E., Hagimoto A., Saito M., Saijo H., Miura H. (2025). Tamoxifen triggers a transcriptional switch from proliferation to differentiation in the circumvallate taste epithelium in mice. Sci. Rep..

[38] Shaner N.C., Steinbach P.A., Tsien R.Y. (2005). A guide to choosing fluorescent proteins. Nat. Methods.

[39] Kishi K., Koyama H., Oka S., Kato A., Sato M., Fujimori T. (2021). Repetitive short-pulsed illumination efficiently activates photoactivatable-cre as continuous illumination in embryonic stem cells and pre-implantation embryos of transgenic mouse. Genesis.

[40] Perley M.J., Kipnis D.M. (1967). Plasma insulin responses to oral and intravenous glucose: studies in normal and diabetic sujbjects. J. Clin. Invest..

[41] Nakagawa Y., Nagasawa M., Yamada S., Hara A., Mogami H., Nikolaev V.O. (2009). Sweet taste receptor expressed in pancreatic beta-cells activates the calcium and cyclic AMP signaling systems and stimulates insulin secretion. PLoS One.

[42] Li Y.S., Meng R.R., Chen X., Shang C.L., Li H.B., Zhang T.J. (2019). Generation of H11-albumin-rtTA transgenic mice: a tool for inducible gene expression in the liver. G3 (Bethesda).

[43] Ahmadzadeh E., Bayin N.S., Qu X., Singh A., Madisen L., Stephen D. (2020). A collection of genetic mouse lines and related tools for inducible and reversible intersectional mis-expression. Development.

[44] Ren W., Aihara E., Lei W., Gheewala N., Uchiyama H., Margolskee R.F. (2017). Transcriptome analyses of taste organoids reveal multiple pathways involved in taste cell generation. Sci. Rep..

[45] Madisen L., Zwingman T.A., Sunkin S.M., Oh S.W., Zariwala H.A., Gu H. (2010). A robust and high-throughput Cre reporting and characterization system for the whole mouse brain. Nat. Neurosci..

[46] Hochedlinger K., Yamada Y., Beard C., Jaenisch R. (2005). Ectopic expression of Oct-4 blocks progenitor-cell differentiation and causes dysplasia in epithelial tissues. Cell.

[47] Beard C., Hochedlinger K., Plath K., Wutz A., Jaenisch R. (2006). Efficient method to generate single-copy transgenic mice by site-specific integration in embryonic stem cells. Genesis.

[48] Wang K., Mitoh Y., Horie K., Yoshida R. (2025). Exploring the role of Ccn3 in type III cell of mice taste buds. J. Neurochem..

[49] Yamase Y., Huang H., Mitoh Y., Egusa M., Miyawaki T., Yoshida R. (2023). Taste Responses and Ingestive Behaviors to Ingredients of Fermented Milk in Mice. Foods.

